# Mechanical Performance of Commercially Available Premix UHPC-Based 3D Printable Concrete

**DOI:** 10.3390/ma15186326

**Published:** 2022-09-12

**Authors:** Carolina Medicis, Sergio Gonzalez, Yezid A. Alvarado, Hermes A. Vacca, Ivan F. Mondragon, Rodolfo García, Giovanni Hernandez

**Affiliations:** 1 School of Engineering, Pontificia Universidad Javeriana, Bogotá 110231, Colombia; amedicis01@javeriana.edu.co (C.M.); sgonzalezb@javeriana.edu.co (S.G.); alvarado.y@javeriana.edu.co (Y.A.A.); vacca@javeriana.edu.co (H.A.V.); 2 Enel Colombia, Bogotá 110221, Colombia; rodolfo.garcia@enel.com (R.G.); giovanni.hernandez@enel.com (G.H.)

**Keywords:** 3D concrete printing, UHPC, anisotropy, mechanical properties

## Abstract

Several recent studies have attempted to formulate printable cementitious materials to meet the printing requirements, but these materials are designed to work with specific printing equipment and printing configurations. This paper aims to systematically develop and perform characterization of a commercially available ultra-high-performance concrete-class material (UHPC) modified to be printable. Four percentages of superplasticizer were used (100%, 94%, 88%, 82%) to adjust the UHPC mixture for 3D-printing requirements. A superplasticizer amount of 88% was considered adequate to meet the requirements. Several fresh and hardened properties of UHPC were measured experimentally: shape-retention ability and green strength were investigated in fresh state, and compressive and flexural strength were evaluated in three loading directions to evaluate the anisotropic effects. Furthermore, the strength of the interlayer bond was investigated. The UHPC developed in this study met the criteria for extrudability, buildability, and shape retention to ensure printability. In comparison with mold-cast UHPC, printed UHPC exhibited superior flexural performance (15–18%), but reduced compressive strength (32–56%). Finally, the results demonstrated that a commercially available UHPC-class material can be used for 3DCP, which possesses all necessary properties, both fresh and hardened.

## 1. Introduction

Three-dimensional concrete printing technology (3DCP) is a construction technique that uses extrusion to print a structure layer by layer without requiring formwork [1,2]. Among its advantages for the construction industry are its excellent productivity and quality, its reduced waste, its increased architectural freedom, and its safety in building environments [3]. Due to layer-by-layer construction without formwork, one of the major challenges of 3D concrete is to ensure that it can pump and extrude into a continuous filament via a nozzle, as well as having high early strength to ensure the maintenance of printed shapes and resist the loads caused by supporting successive concrete layers without large deformations or collapse. In addition, the concrete has to achieve strong and durable mechanical properties for an adequate service life [4,5,6].

Conventional commercially available concretes are considered unsuitable for 3DCP because of their fresh state characteristics, which require formworks. For cementitious materials to be printable, they should have low viscosity for uninterrupted flowability during pumping and extrusion, as well as high yield stress to maintain their shape during and after printing. In this regard, different commercial concrete materials could be used to build elements with different mechanical requirements if the necessary requirements are met in a fresh state [7].

As a material for 3D printing, ultra-high-performance concrete (UHPC) may be more suitable than normal and high-strength concrete because of its superior strength. However, even though UHPC has been commercially available for over a decade, the knowledge of its use in 3D printing is just beginning to become more widely available. An example of a typical UHPC mix design would include Portland cement, silica fume (SF), quartz powder (QP), quartz sand (QS), polycarboxylate-based high-range water reducer (HRWR) and steel fibers [8,9]. Due to its high mechanical properties and durability, UHPC has been used in a variety of applications, including those that require adapting its rheology [10]. For example, on sloped surfaces of casting, UHPC with relatively high yield stress should be used to prevent material flow from elevated to lower locations. Instead, casting UHPC elements requires a UHPC mix of relatively low yield stress to ensure high filling ability and reduce consolidation energy. The rheology of UHPC can be adapted by modifying HRWR content. The HRWR reduces water consumption and improves the workability, flow, and strength of the concrete [11]. In addition, it allows for an increase in the plastic viscosity and cohesion of printing mixtures [12]. Based on the above, adjusting the HRWR dosage may be possible for the development of UHPC mixtures that meet 3DCP requirements.

With regard to the mechanical performance in fresh and hardened state of the 3DCP material, researchers worldwide have developed methods to measure these characteristics. Measurement of early age concrete mechanical strength is one of the ways to find the stiffness and strength development of printable materials. As it is a new technique, some studies have investigated it through compressive strength tests at an early age [11,13,14], and the shape-retention ability determines its capability to retain the shape of the printed layers to support consecutive layers [3,15,16]. In terms of mechanical performance, unlike cast specimens, printed structures exhibit anisotropic and isotropic properties [17,18]. Therefore, it is crucial to evaluate the mechanical properties in different loading directions through compressive and flexural tests [19]. The anisotropy of 3DCP has been studied by several researchers. According to their findings, 3DCP is more anisotropic and has less strength in the direction parallel to the vertical axis, compared to the other two directions [19,20,21].

The objective of this research was to evaluate fresh and hardened properties of commercially available premix UHPC-based modified for printing. For this purpose, a number of mixtures were designed to find an optimum dosage of HRWR for 3D printable UHPC. Subsequently, a systematic experimental program was performed to study the fresh properties such as slump, shape retention and green strength. Finally, mechanical performance such as compressive and flexural strength of 3DCP related to the printing direction, were systematically characterized.

## 2. Materials and Methods

### 2.1. Materials and Mix Preparation

The concrete used in this study is a UHPC-class material produced by Cementos Argos. Manufacturers supplied three primary components for this product: a preblended powder mix containing all of the solids (Portland cement type III, silica fume, calcium carbonate and the fine aggregate of a particle size under 600 μ m), superplasticizer HRWR agent based on polycarboxylic (ASTM C494 requirements for Type F), and steel fiber reinforcement, which was not used in this study.

The mixing procedure was as follows: firstly, water and HRWR were mixed for 1 min; secondly, 50% of the dry cementitious and sand was added and mixed for 4 min; then, 25% of the dry cementitious and sand was added, and mixed for 10 min; finally, the rest of the dry cementitious and sand was added, and the mixture was mixed for another 20 min. About 35–40 min was required for batching and mixing. Due to low flowability being a prerequisite for achieving buildability and maintaining shape and position after deposition of 3D concrete printing, the initial UHPC dosage was modified. According to previous research, to control the behavior of concrete printing materials, the water-to-binder ratio (w/b) can also be modified [22]. Hence, a low concrete slump can be achieved by reducing the amount of water or water reducers.

To adjust the UHPC mixture with respect to 3D printing in terms of flowability, extrudability and buildability, three low-consistency samples were prepared with a superplasticizer content reduction of 6%, 12%, and 18%. The mini-slump test or the spread table test was used to quantify the flowability of the mixtures being studied, and it is associated with the dynamic yield stress, which can affect the pumpability and extrudability of the mixtures in 3DCP [5,23]. The measurement of the slump flow is the average diameter of the horizontal flow measured in two perpendicular directions after lifting the cone (cone size is diameters of 70–100 mm, and height of 50 mm).

**Figure 1:**
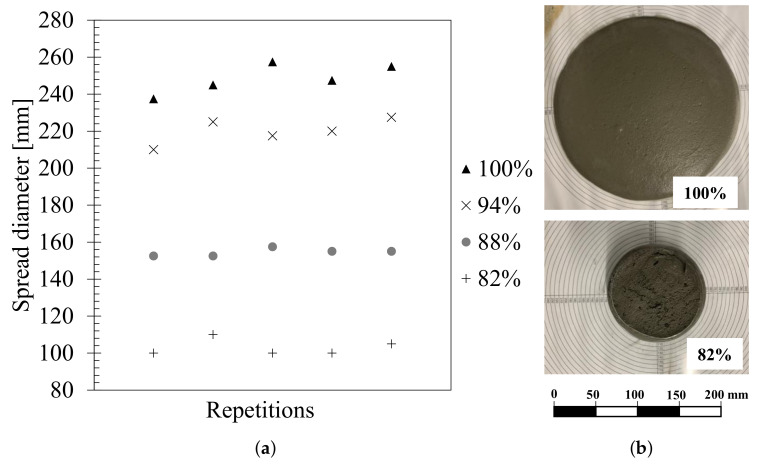
(**a**) Effect of HRWR on slump flow diameter, and (**b**) Spread diameter with HRWR content of 100% and 82%.

Generally, authors report that their 3DCP setup produces the most desirable print quality and buildability with a slump value of less than 110 mm [3,24]. Nevertheless, some authors have said a slump value major is needed. Arunothayan et al. [3] recommended a slump value of 119 mm, Ma et al. [25] considered a slump value between 132–188 mm, and Zhang et al. [11] found that such a value should be 190–252 mm. As well as in other 3DCP studies, the mixture was selected based on the required criteria of fluidity, pumpability, extrudability, and buildability. It was selected via qualitative methods, including visual inspections to evaluate the surface quality and dimensional consistency [4,5,6]. The change in slump flow diameter for UHPC mixtures is shown in Figure 1a,b. As a result, it was found that the UHPC mixture with 88% HRWR with a slump value between 147–155 mm made it possible to pump and extrude properly 3D printing concrete due to its fluidity. Additionally, the shape of the material can be retained after it leaves the nozzle. According to the above, a suitable adjustment of cement with HRWR results in 3D printing concrete ink showing excellent fluidity during the printing and excellent standing behavior during a static state.

### 2.2. 3D Concrete Printing Equipment

**Figure 2:**
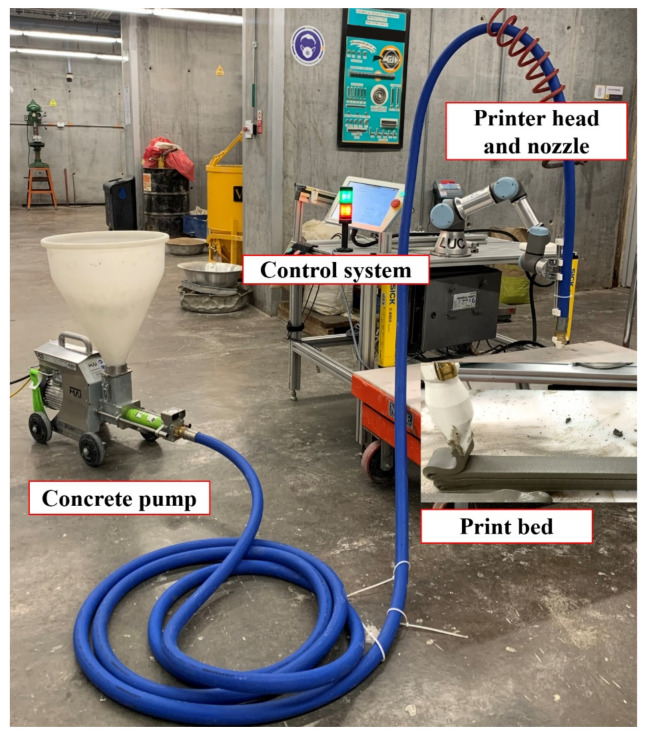
Printing system (progressive cavity pump-MAI®2PUMP-PICTOR, and robotic arm-UR3).

The printing system was a lab-scale setup. The setup comprises a progressive cavity pump (MAI®2PUMP-PICTOR) and a six-axis industrial robot (UR3), as shown in Figure 2. The concrete was mixed and placed into the hopper to be pumped into a hose (10.0 m long and Ø25.4 mm) attached to the robotic arm and connected to a custom-designed nozzle with dimensions of 40 mm in width and 10 mm in height. All the nozzles were printed using acrylonitrile butadiene styrene (ABS) thermoplastic filaments. During the printing process, the pump flow rate and the printer speed were calibrated to ensure continuous flow and good stability and shape of printed layers. The printing speed was 2100 mm/min at a constant pump flow rate of 0.7 L/min.

### 2.3. Test Methods

#### 2.3.1. Mechanical Properties of Fresh 3DPC

The common test methods for the fresh properties of normal concrete cannot accurately capture the properties of 3DPC. Accordingly, the methods commonly used by researchers to measure fresh properties in 3DCP are as follows. Mechanical behavior at early age in fresh concrete must be able to support its own weight and as well as the weight of the layers above it, and limit deformations. Therefore, three quantitative fresh tests were conducted to assess its mechanical behavior at an early age: mini-slump, shape retention, and green strength. Due to the UHPC being sensitive to temperature and relative humidity, a mini-slump test was used to ensure that the mix has the required flowability and workability.

**Figure 3:**
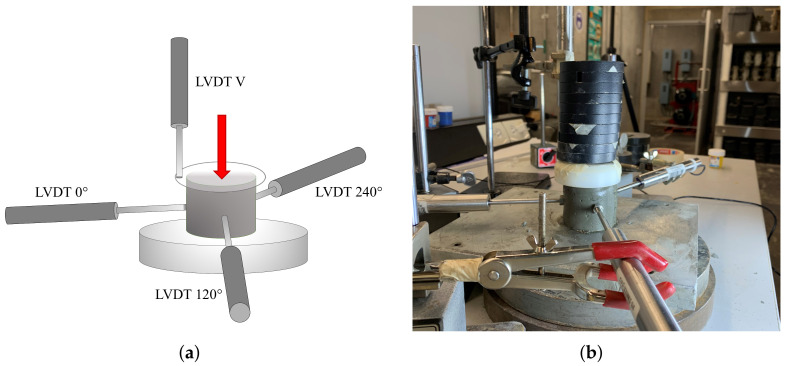
(**a**) Schematic drawing of the shape retention test, and (**b**) Shape retention test laboratory setup.

The ability of the fresh mixture to hold its shape after extrusion (shape retention-SRA) was determined. For the test, the fresh mixture was poured into a 60 mm diameter and 60 mm height plastic cylinder and vibrated for 10 s. After two minutes, the cylinder was unmolded and the SRA was measured using linear variable differential transformers (LVDT) to quantify the change in diameter and height, at four points of the fresh specimen. The springs of the LVDT were removed and a nut was placed in each one of them to assure a flat contact surface with the sample and to avoid penetration of them into the fresh specimens. The measurement locations are shown in Figure 3a, including the vertical displacement (LVDT V) at the top of the specimen, and the horizontal displacement measured by three LVDTs located at diametrically locations at 0, 120 and 240 degrees (LVDT 0, LVDT 120 and LVDT 240). To simulate the printing time interval between layers, referred to as delay time, the top of the fresh mixture was gradually loaded with circular steel plates of 200 g weight every two min until its failure (For uniform pressure distribution, a plastic plate was placed on top), as shown in Figure 3b. It method was used by previous studies to investigate the shape-retention-ability of the fresh printing materials [3,12,15]. Arunothayan et al. quantified the SRA using Equations (1) and (2) [3]:

(1) $$SRA_{d}=\frac{d_{L}}{d}$$

(2) $$SRA_{h}=\frac{h_{L}}{h}$$

where, $d_{L}$ and $h_{L}$ are the diameter and height of the sample at a given load *L*, and *d* and *h* are the diameter and the height before load application. $SRA_{d}$ and $SRA_{h}$ determine the dimensional variations with respect to the diameter and the height of the fresh specimen, respectively.

**Figure 4:**
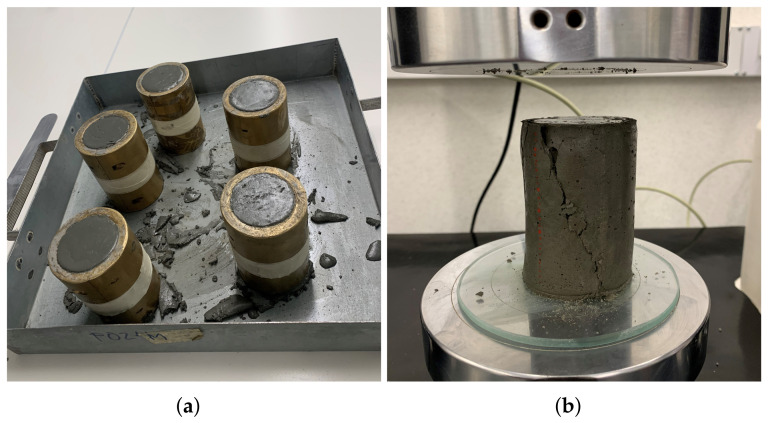
(**a**) Molds for UUCT, and (**b**) Fresh specimen after the green strength test at 120 min.

In addition, the mechanical behavior of the bottom layers is severely critical during the printing period due to the time required to build every component [26]. One approach to monitoring the development of very early strength is to measure the uniaxial unconfined compressive strength test (UUCT), according to ASTM D2166-91, of fresh concrete at different ages [26,27]. In 3DCP, the UUCT is also called green strength [5,14]. The specimen dimensions were 50 mm in diameter and 100 mm in height. The material was molded into cylindrical molds and compacted two times for 10 s on a 30 Hz vibration table to realize a homogeneous sample (Figure 4a). After 10 min, the samples were carefully unmolded and tested at the corresponding age. The green strength test was conducted at 30 mm/min using an electromechanical universal testing machine, and five separate samples of the same batch were prepared and tested at 0, 15, 30, 45, 60, 90, 120, and 180 min of rest (Figure 4b).

#### 2.3.2. Mechanical Properties of Hardened 3DPC

**Figure 5:**
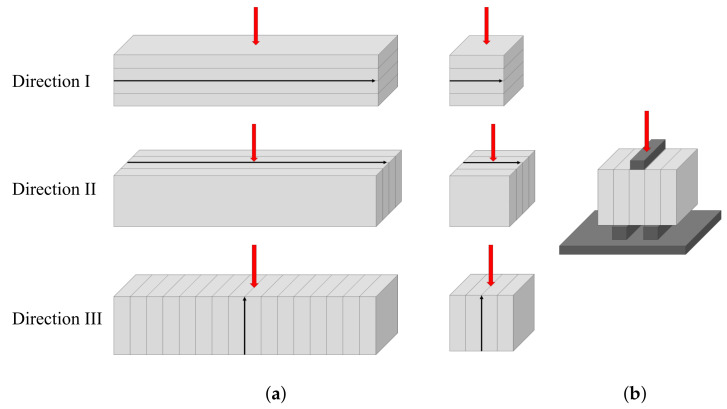
(**a**) Schematic drawing of the three loading directions for compression and flexural specimens, and (**b**) schematic drawing of the custom-made apparatus used to the interlayer shear strength test.

**Figure 6:**
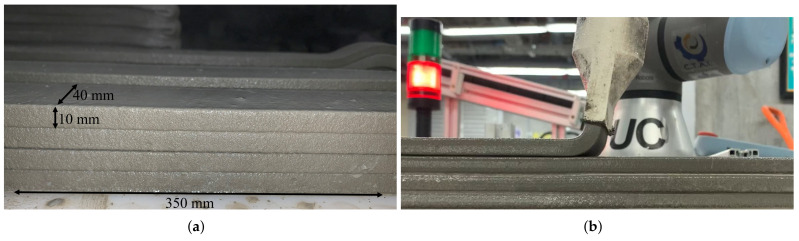
(**a**) 3D printed filaments for mechanical tests. (**b**) UHPC being printed.

Load direction was determined to explore the anisotropy of printed composites, as shown in Figure 5a: in direction I, the force was applied perpendicular to the printing direction; in direction II, the force was applied parallel to the printing direction; and in direction III, the force was applied parallel to the interfaces of the layers. Compression and flexural specimens with dimensions of 40 × 40 × 40 mm and 160 × 40 × 40 mm, respectively, were sawn from the 350 × 40 × 40 mm printed filaments (Figure 6). Five specimens were tested for each test direction according to ASTM C109 and ASTM C348 for compression and flexural tests, respectively.

Furthermore, due to the layer-by-layer printing and the open time effects, the inter-facial shear strength is a critical parameter of weakness [28]. A custom-made apparatus was used to measure the inter-facial shear strength of 3D printed concrete specimens, as shown in Figure 5b. The size of the cut specimens was 40 mm in height and 50 mm in width, and five specimens were tested at a displacement rate of 1 mm/min after 28 days of curing. The specimen is placed in the center of the compression plate of the machine.

## 3. Results and Discussion

### 3.1. Fresh Properties

**Figure 7:**
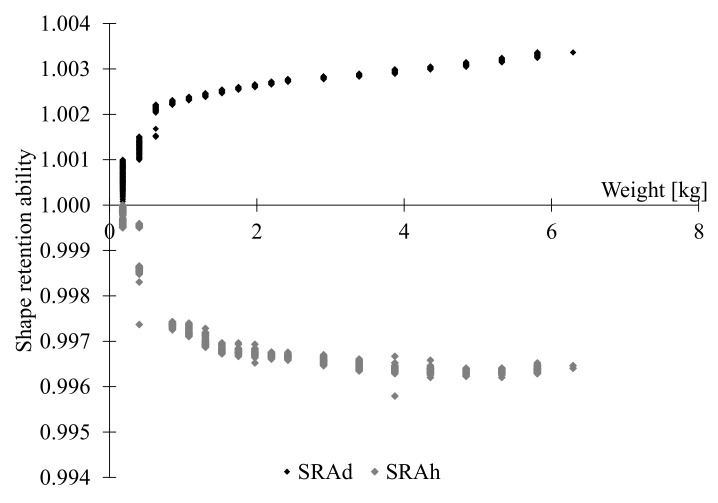
Shape retention ability indices.

**Figure 8:**
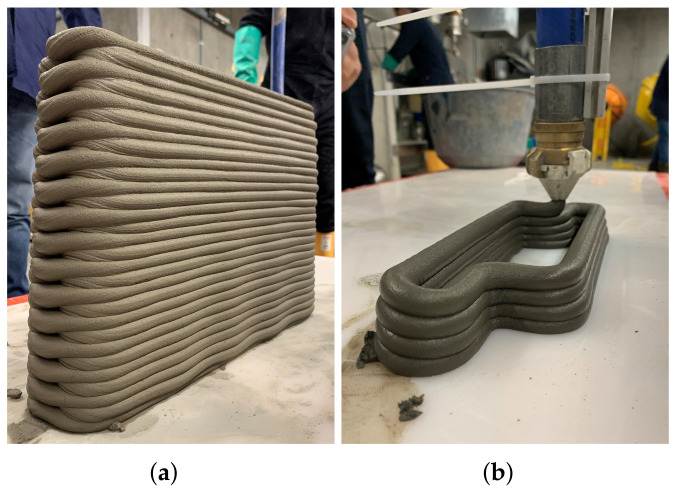
(**a**) 3D printed wall with UHPC, and (**b**) UHPC being printing.

Figure 7 shows the SRAd and SRAh indices of the UHPC as a function of the weight. Both the vertical and horizontal indices shows a small increment in the indices with increasing deformations, up to a certain plateau. This means that fresh mixture had almost zero slump, which indicates a high ability to retain the shape. The results are significant, as they indicate behavior desirable for 3DCP processes based on extrusion. In addition, the print tests demonstrated adequate shape retention using different nozzle shapes (rectangular, as shown in Figure 6, and circular, as shown in Figure 8). The behavior was reflected as small plastic deformations shown as a decrease in filament height as a function of extruded layers.

**Figure 9:**
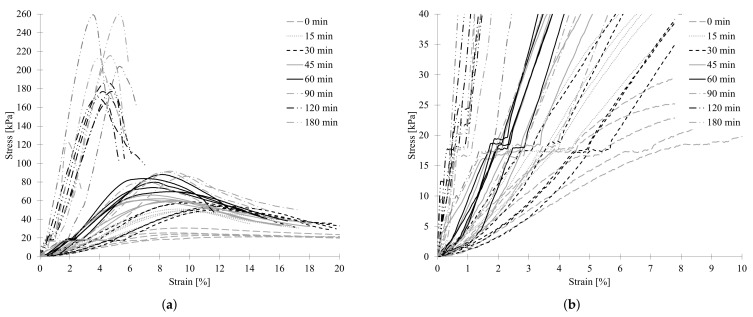
(**a**) Green strength vs. vertical displacement diagrams of the UHPC on fresh state at different resting times, and (**b**) cracks occurrence.

The stress–strain response of UHPC is presented in Figure 9a. Vertical strain was calculated by dividing vertical displacement by sample initial height, and as a result, plastic behavior was observed. Compression stress was obtained by dividing the axial load by the cross-sectional area, which was calculated according to ASTM D2166-91.

It was observed that the response of behavior of the fresh specimens is similar to that reported in Refs. [1,13,29]. For each specimen, a linear increase in vertical displacement as the load increases initially is evident. The results indicate that strength and stiffness increased significantly in early time. There was a noticeable change in behavior at an early age, from 0 to 90 min and 90 to 180 min. In the younger specimens, the displacement increased as the load increased until a plateau was reached. On the other hand, in the case of older specimens, once the peak was reached, the load decreased up to a certain limit. The differences in behavior are attributed to different material failure mechanisms. For example, younger specimens fail by expanding in the lateral direction as the vertical deformation increases, also known as the barrelling effect. Older samples fail with a failure plane with lower deformations.

The average strength increased from 26.75 kPa at 0 min to 84.47 kPa at 60 min to 181.70 kPa at 120 min, and 239.41 kPa at 180 min. It is observed that the early age strengths are significantly higher than the values reported in the literature for custom-designed printable concrete mix. Wolfs et al. [30] used a mix of Portland cement (CEM I 52.5 R), siliceous aggregate with a maximum particle size of 1 mm, limestone filler, additives, rheology modifiers, and a small amount of polypropylene fiber. They reported an average compression strength of the youngest specimen equal to 6.37 kPa, which increased to an average of 18.93 kPa at 90 min. These values are about 78% lower than those found in this study. Zhu et al. [1] investigated 3D concrete printing of permanent formwork for concrete column construction. The highest peak stress measured was 51.5 kPa at 90 min, which is still 44% lower than that measured by this study at the same age.

On the other hand, during the first part of the tests, strain hardening was observed in the samples at low load (about 17 kPa), as shown in Figure 9b. Lee et al. [22] attributed the phenomenon to the viscous resistance between particles until critical stress was reached due to the greater contact force between cement particles due to the lack of water. Gong et al. [31] described the phenomenon in three stages. In the first stage, the load was low enough that the dispersion medium and particles could easily spread into the surrounding area. During the second stage, even though the load increased, almost no particles were in contact with each other. At this time, the force was caused by liquid viscous force between the particles. At the third stage, a network structure gradually formed as particles contacted each other. As a result, particles could not spread, so, when the suspensions reached the boundary, they jammed, causing cracks and increasing forces. Finally, at a critical force level, specimens were bulging and then cracked vertically, without exhibiting elastic behavior.

### 3.2. Hardened Properties

**Figure 10:**
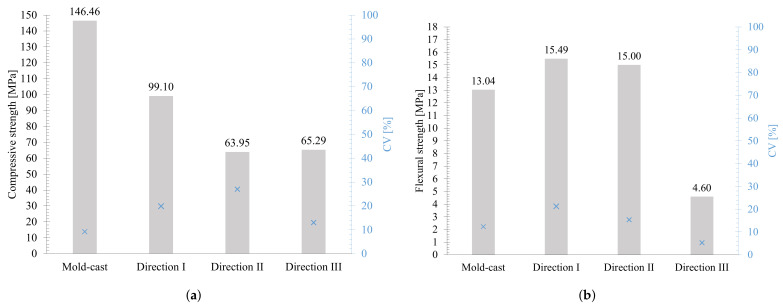
(**a**) Average compressive strength of the UHPC, and (**b**) Average flexural strength of the UHPC. The coefficients of variation (CV) are reported on the blue axis.

Figure 10a presents the 28-day average compressive strengths of the UHPC mixture (the optimum 3D printable mixture-88%) printed for three different load directions. As with other 3D printed concretes used by other researchers, the UHPC mixture also exhibited anisotropic behavior in response to the loading directions [12,16,32]. According to the results, direction I has the highest compressive strength value compared to the other two directions. Directions II and III are weaker, with an average reduction of 35%. This may be attributed to layers compacting as the material is extruded, resulting in a more dense structure in the direction of printing [33,34]. Moreover, the conventional mold-cast UHPC compression strength reference value is 146.46 MPa, which means that 3D-printed specimens had a reduction of 32–56%. This could be explained by a possible increased porosity of the printed UHPC due to lack of vibration during printing process, compared to the mold-cast UHPC. However, extrusion pressure contributes to greater longitudinal compaction of the material. Consequently, the maximum compressive strength is found in the longitudinal direction [3]. Compared to other 3D printable cementitious materials with and without fibers, the experimental results indicate an average compressive strength between 63–16% higher [12,16,21,35,36].

As with the compression strength results, anisotropic behavior was observed for the flexural strength tests in the three different load directions. Average flexural strength results show a more pronounced difference in directions II and III, and average strength reduction of 13.8% and 73.6%, respectively, compared to the direction I was found (Figure 10b). Additionally, it was observed that the specimens tested in direction III had lower variability (CV) than those tested in the other directions, which may be due to a more brittle failure mode [33]. Moreover, the conventional mold-cast UHPC flexural strength reference value is 13.04 MPa, which means that 3D-printed specimens tested in direction I and II had an increase of 18 and 15%, respectively, whereas in direction III, they had a reduction of 64%. Printed specimens (direction I and II) have a significantly higher flexural strength than mold-cast specimens, contrary to the results presented in the compressive strength. This can be attributed to the loss of water during the extrusion process, which increased the strength of the material [37]. In addition, this can be explained by the confinement caused by the extrusion and the overlay layers. According to the experimental results, the 3D printable cementitious materials with and without fibers showed about 50% higher average flexural strength in the direction of printing [16,36].

**Figure 11:**
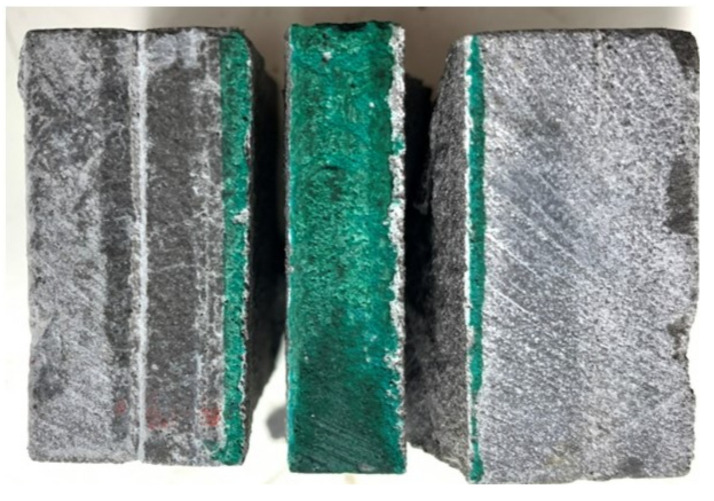
Interfacial shear strength of the UHPC.

The flexural strength of the tested specimens depends on the interlayer bond strength between layers (especially in direction III). Therefore, it is necessary to study the behavior between layers. Figure 11 presents the 28-day interlayer strengths vs. vertical mid-span displacement curves (a), and the failure mode pattern (b). The average value of interlayer strength was 4.51, and the CV was 0.07, which was similar to the samples tested under flexural loading in the direction III. Results are consistent, with low variability, and an average strength difference of 2.2%.

**Figure 12:**
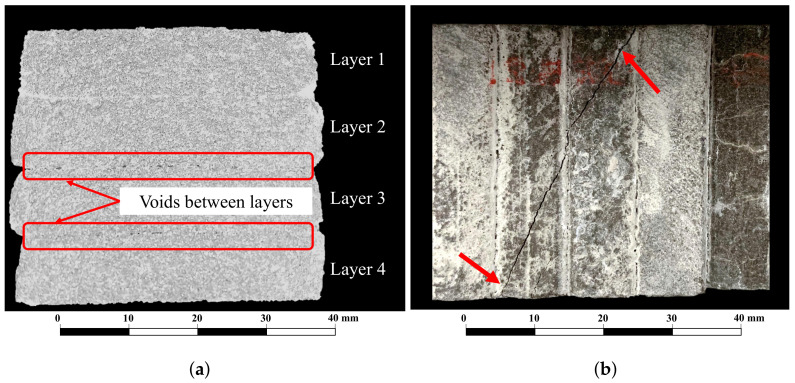
(**a**) Observation of voids between layers in a 3D-printed UHPC specimen, and (**b**) specimen with a crack that did not propagate through the interface.

Figure 12a shows how the voids between the filaments could lead to weak planes and contribute to the formation of cracks under vertical loads [35]. As a result, vertical weak planes in direction I may induce weaker mechanical behavior (strength and stiffness) than in the other two directions, leading to anisotropic behavior, which is consistent with the results of compressive and flexural tests.

On the other hand, there were cases when cracks did not propagate through the interface but in the adjacent layer, as shown in Figure 12b. Nevertheless, they were excluded from this research because the study of crack propagation patterns is not within the scope of this study. It also was reported by other authors. Murcia et al. [35] argue that pressure exerted during the printing process reduces the overall porosity of 3D printed concrete. This argument was also backed up by Panda et al. [34], who added that the flow of the overlay layer caused the two layers to intermix to a certain extent due to the stresses that were generated by the flow.

Finally, it was quantitatively evaluated the mechanical–anisotropic behavior of printed UHPC by an equation that represents the anisotropy coefficient, using Equations (3) and (4) [36,38].

(3) $$\begin{array}{c} f_{3D}=\frac{\sum_{n=1}^{i}f_{xn}+\sum_{n=1}^{i}f_{yn}+\sum_{n=1}^{i}f_{xn}}{3i} \end{array}$$

(4) $$\begin{array}{c} I_{a}=\frac{\sqrt{{\left(f_{xi}-f_{3D}\right)}^{2}+{\left(f_{xi}-f_{3D}\right)}^{2}+{\left(f_{xi}-f_{3D}\right)}^{2}}}{f_{3D}} \end{array}$$

where $f_{xn}$ , $f_{yn}$ and $f_{zn}$ are the average strength of printed specimens loading in different directions (*x*, *y*, and *z*, respectively), $f_{3D}$ refers to the average strength of all loads directions; $f_{xi}$ , $f_{yi}$ , and $f_{xi}$ refers to the *i*-th strength of the printed specimens subjected to the loading direction; and $I_{a}$ indicates how printing affects the mechanical properties of printed concrete. A higher value of $I_{a}$ indicates more influence on the mechanical anisotropy because of the printing process. As the value approaches zero, effects caused by the printing process can be ignored, as will the mechanical anisotropy.

The $f_{3D}$ and $I_{a}$ values of compression strength were 76.11 MPa and 1.09, respectively, and the $f_{3D}$ and $I_{a}$ values of flexural strength were 11.70 MPa and 21.41, respectively. The UHPC showed a low coefficient (close to zero) in compression performance, but a high coefficient in flexural performance, indicating significant mechanical anisotropy because of the printing process. Nevertheless, the anisotropic behavior of the printed concrete can be likened to the anisotropic behavior of the wood, considering that at the macroscale, the wood is formed by multiple layers containing earlywood and latewood rings. Despite the anisotropy, there is an extraordinary amount of consumption of wood, making it one of the most important materials, and it is even used structurally as columns and beams. It is therefore essential that the mechanical properties of a structure be evaluated in different loading directions through compressive and flexural tests so that the best structural performance can be achieved.

## 4. Conclusions

This study demonstrates the systematic experimental development and characterization of fresh and hardened properties of a commercially available UHPC-class material modified for a 3DCP construction technique. It evaluated fresh properties such as mini-slump, shape retention, and green strength, as well as hardened properties such as compressive and flexural strengths of 3DCP related to printing direction. In addition, the mechanical–anisotropic behavior of the printed UHPC was evaluated. Based on the above results and discussion, the main findings of this study are:

- By reducing the superplasticizer dosage in fresh UHPC mixture, it was possible to achieve the requirements for a commercially available UHPC-class material printable.
- The results in the fresh state indicated high buildability was obtained with minimal deformations, as well as high strength at early ages.
- The mechanical performance showed anisotropic behavior. The highest flexural strengths were achieved when force was applied perpendicular and parallel to the printing direction. In both directions, performance was similar, but flexural strength was lowest when applied parallel to layer interfaces. In contrast, the printed UHPC samples showed decreased compressive strength. In the direction perpendicular to the printing direction, the compressive strength was highest, while in the other two directions, it was similar. In addition, the compressive strength of the printable specimens was lower than that of the mold-cast specimens. Flexural strength, however, was higher in printed specimens than in mold-cast specimens. Finally, UHPC performs well in both fresh and hardened states, making it possible to build thinner 3D printed elements than with other 3D printable concretes.
- In this report, one of the four basic components of the UHPC was modified (binders, aggregates, water, and additive) to produce a commercially available premixed UHPC-based printable. Nevertheless, it is one of the possibilities. To determine a dosage mixture that results in optimal behavior, more research will be conducted in the future by modifying different components.

## Data Availability

Not applicable.
